# Different Approaches to Study Molecular Blueprint and Biological Behavior of Brain Tumors: Editorial to the Special Issue “Advances in Molecular Genetics of Brain Tumors”

**DOI:** 10.3390/ijms24020948

**Published:** 2023-01-04

**Authors:** Nives Pećina-Šlaus, Ivana Jovčevska

**Affiliations:** 1 Laboratory of Neuro-Oncology, Croatian Institute for Brain Research, School of Medicine University of Zagreb, Šalata 12, 10000 Zagreb, Croatia; 2 Department of Biology, School of Medicine, University of Zagreb, Šalata 3, 10000 Zagreb, Croatia; 3 Center for Functional Genomics and Biochips, Institute of Biochemistry and Molecular Genetics, Faculty of medicine, University of Ljubljana, Zaloška cesta 4, 1000 Ljubljana, Slovenia

Cancer remains one of the leading causes of mortality worldwide. In this context, brain tumors are also characterized by poor prognosis and short survival [1,2]. Brain tumors demonstrate the great heterogeneity and inherent molecular variability of tumor cells even within a specific subtype. Therefore, for precise diagnosis and successful treatment it is important to understand their biology, which, at this point, remains largely unexplained. Understanding, preventing, and treating brain tumors are among the most important ongoing challenges of neuro-oncology and a crucial public health issue.

The aim of the Special Issue “Advances in Molecular Genetics of Brain Tumors” was to present a collection of selected relevant papers in the rapidly expanding field of brain tumor genetics. The topics covered molecular genetics, signaling pathways governing the mechanisms of tumor formation and invasion, DNA methylation, predictive biomarkers, diagnostic improvement, potential therapeutic targets, metastasis and recurrence, as well as the difference in the therapeutic response to specific clinical subtypes. In this issue, we published a total of 14 papers, particularly 10 novel innovative studies on the genetics of brain tumors, as well as 1 communication and 3 reviews providing perspectives that can shed light on novel research directions. The contributing authors report a range of different and versatile molecular studies, but also offer insight into the state-of-the-art progress in several critical reviews.

The original work on the genetics of brain tumors consists of diverse studies ranging from profiling of glial cancers pathway-related genes [3] on one end, to investigating the migratory properties of glioblastoma cells [4] and proteins involved in the process [5], and identification of proteins crucial for stemness and self-renewal of glioblastoma cells [6] on another end. In more detail, in the comparative study by Majercikova et al. [3] the authors performed transcriptomic profiling of cancer pathway-related genes by combining the PCA method and multi-criteria decision making in the analysis of gene expression. They observed changes in the expression of 26 genes compared to the average expression value of three different controls belonging to various pathways including cellular senescence, metabolism, angiogenesis, apoptosis, DNA damage and repair, epithelial to mesenchymal transition, and telomeres and telomerase. Migratory glioma cell properties were examined in two studies: one by Odrzywolski et al. [4] analyzed the correlation of doublecortin (DCX) expression and glioblastoma cell migration using immunohistochemistry and single-cell RNA-seq. Along with DCX, the analysis also included the expression patterns of Nestin (NES) and Oligodendrocyte lineage transcription factor 2 (OLIG2). The findings indicated that there was a set proportion of cells expressing DCX/NES/OLIG2, regardless of treatment, guided by tumor plasticity.

The second study on migratory properties by Toedebusch and colleagues [5] is about the involvement of microglia-derived olfactomedin-like 3 (Olfml3) in glioma progression. Toedebusch et al. performed CRISPR-Cas9-mediated Olfml3 gene editing in N9 cells and clearly demonstrated that this extracellular matrix protein promoted glioma cell migration and invasion. Other essential mechanisms examined were stemness and self-renewal, as reported by Wu and coauthors [6]. In their study, the authors reported that the expression of BIRC3, a member of the IAP family of proteins that inhibit apoptosis, promotes glioblastoma stemness and tumorigenicity of glioma stem cells (GSCs) through inactivation of BMP4 signaling pathway. Elucidating the mechanisms of GBM stemness reprogramming and adaptation, which is believed to be a primary cause of therapeutic failures, is very important for advancing our understanding of glioblastoma resistance to therapy (Figure 1).

Moreover, studies also reported tumorigenesis mechanisms controlled by the circadian gene cry [7]. Circadian rhythm regulation [8] has only recently been introduced in cancer research. However, the deregulation of circadian genes in tumors has been recognized as important. Using an in-house *Drosophila* model, Jarabo et al. reported a novel role of the light-regulated protein Cry that acts as a core component of the circadian clock. The model uses the system of co-activation of EGFR and PI3K signaling pathways in Drosophila glial cells.

The mechanisms affected by temozolomide in pediatric glioblastoma was investigated by Damanskienė et al. [9]. The authors tested the differences in efficacy of temozolomide doses between PBT24 and SF8628 cell lines of high-grade pediatric glioblastoma xenografts in a chicken chorioallantoic membrane (CAM) model. The study stressed the importance of personalized therapy for glioblastoma, emphasizing that it should be specifically tailored to the pediatric population.

The importance of metabolic dysregulation has long been recognized as a driving force of cancer, particularly glioblastoma. In their contribution, Franceschi et al. addressed a very important aspect of glioblastoma progression: the involvement of a metabolic pathway parallel to glycolysis, specifically the pentose phosphate pathway (PPP), which plays a critical role in sustaining cancer-cell survival and growth [10]. The authors evaluated the role of sedoheptulose kinase (SHPK), an enzyme involved in the nonoxidative arm of the PPP, by conducting a functional enrichment analysis using microarray data on SHPK expression in glioblastoma patients. Further, they evaluated the effects of SHPK overexpression in three different glioblastoma cell lines. Their results showed that the increased SHPK expression was significantly correlated with a worse glioblastoma prognosis.

Moreover, two studies tackled RNA interference in glioma. Namely, the need to identify miRNAs as specific non-invasive biomarkers for the prognosis of glioma is highlighted in the work of Levallet et al. [11]. The authors reported that the expression level of a panel of seven pro-angiogenic and/or pro-hypoxic miRNAs (has-miR-200b-3p, -200c-3p, -210-3p, -126-5p, -221-3p, -424-5p, and -451-5p) was affected in patients with glioma and related to the glioma histology grade. The results suggest that pro-angiogenic and/or pro-hypoxic miRNAs can be used as tools for monitoring patients, specifically with IDH-mutated low-grade tumors, since they are easily measurable in plasma. In a different study, Clausing et al. [12] examined the effect of IDH1^R132H^ mutation on the redox system in a CRISPR/Cas edited glioblastoma model and compared them with IDH1 wild-type (IDH1wt) cells. This model is suitable for portrait IDH1^R132H^-dependent alterations in tumor cell metabolism. In the study, they also showed an increase in NAD+ in IDH1^R132H^ glioblastoma cells compared to IDH1^wt^. Their findings underline the therapeutic potential of targeting the NAD+ synthesis pathway, but authors recommend caution for small-molecule inhibitors.

In the Special Issue, we also included one study about the importance of FET PET/CT scans [13]. With the purpose of treatment improvement, Skoblar Vidmar et al. tested the performance of O-(2-[^18^F] fluoroethyl-)-L-tyrosine (^18^F-FET) PET for the differentiation between glioma patients based on IDH mutational status. The enzyme isocitrate dehidrogenase (IDH) is a biomarker that improves diagnostic accuracy, but also influences the response and the course of treatment, and thus, overall survival [14]. This is the first reported study that assessed the diagnostic performance of different ^18^F-FET PET segmentation approaches for differentiation between treatment-related changes (TRC) and true progression (TP). The neuro-oncological therapy can lead to the development of TRC that mimic TP and distinguishing TRCs from TP in treated patients remains a challenge in glioma cases, since both share similar clinical symptoms and imaging characteristics [14]. In their contribution, Skoblar Vidmar and co-authors stressed the importance of molecular biomarkers that have clinicopathologic utilities.

One study focused on a different group of most common primary brain tumors i.e., meningiomas [15]. Bukovac and coauthors investigated the role of DVL1 that is the central mediator of Wnt signaling pathway. The results revealed that the central PDZ region of DVL1 gene harbored frequent mutations. The study further showed that the samples containing mutations in the PDZ domain expressed significantly less DVL1 protein and that the nuclear expression of DVL1 was significantly correlated with a higher expression of active β-catenin (*p* = 0.029) and a higher meningioma grades (*p* = 0.030). Their genomic instability, sequencing and immunohistochemistry results indicate that Wnt signaling is activated in meningioma and that DVL1 could potentially represent a good biomarker for meningioma progression.

To obtain a broader view of the field, we published three review papers that provided comprehensive and thoroughly updated critical standpoints on several topics such as targeted therapies for vestibular schwannomas [16], as well as molecular biomarkers [17] and precision oncology for complex diseases such as glioblastoma [18]. Tamua and Toda prepared an in-depth update of the currently available knowledge on the molecular biology of vestibular schwannomas and its relevance to treatment. The importance of the tumor microenvironment, inflammation, and stress reaction in the development and progression of vestibular schwannomas is also critically surveyed. The review discusses a range of therapeutic approaches, from surgery and radiation therapy to gene therapy. Finally, the authors indicate that tumor-microenvironment-targeted therapy may also be supportive and recommend multimodal therapy for patients with refractory vestibular schwannomas. Next, Sareen et al. [17] conducted a systematic review and performed meta-analysis of key molecular biomarkers that have been investigated for their predictive value in recent glioblastoma clinical trials. Analysis of the prognostic significance of IDH1 mutation showed significantly better overall survival in patients with IDH1 mutation. Meta-analysis including 575 glioblastoma patients presenting with either amplification or high expression of EGFR gene did not reveal prognostic significance, which the authors contributed to limited patient numbers; they recommended more homogeneous studies on larger patient cohorts. Lastly, Panovska and De Smet [18] surveyed the current knowledge on therapy approaches for complex and heterogeneous diseases such as glioblastoma that display formidable inter- and intra-tumoral heterogeneity. The authors appeal on personalized and distinct therapeutic approaches in order to achieve clinical benefits since the clinical trials of the past 20 years have failed to improve the outcome for the vast majority of glioblastoma patients. The review highlights the need for procedures that can precisely select the appropriate patients who could benefit from the given therapy, but also to the drug sensitivity of specific tumor cells of a particular patient. The authors discuss the current state of the art of transforming technologies, tools and challenges for functional precision oncology, and conclude that that the personalization of cancer medicine is the way to tackle this disease.

In conclusion, in order to better understand the biology of brain tumors and ultimately improve diagnostic and therapeutic approaches, this Special Issue intends to elucidate the diversity of problems behind brain tumors and their potential solutions. This is also nicely illustrated with the diversity of methodologies used in the papers published in our Special Issue. As brain tumor incidence increases with age, with the growing numbers of elderly population, the number of patients is expected to rise. Many cases of brain tumors are characterized with discouraging prognosis. The treatment resistance and the possibility of recurrence is the cause of poor survival, especially for the glioma branch [19].

To improve patient outcome, numerous novel ideas and alternative approaches are constantly being explored by the research community. Molecular testing in modern cancer diagnostics, combined with the development of personalized therapies, is the main avenue for the successful outcomes and progress. However, there is still room for improvement, which demands that we search for novel crucial molecular players and development of new concepts about the initiation and progression of brain tumors. As reported in this Special Issue, brain tumors are investigated on various cellular levels with the ultimate goal of greater patient benefit. We hope that the themes and fields covered in this Special Issue will attract readers from the broader scientific community, contribute to expanding our knowledge about the biology of brain tumors, and inspire further studies that will improve the diagnosis and clinical management of brain tumors.

Lastly, as Guest Editors, we would like to gratefully acknowledge the input of all the authors of both original research articles and review papers, and their contribution to this relevant and valuable research topic.

## Figures and Tables

**Figure 1:**
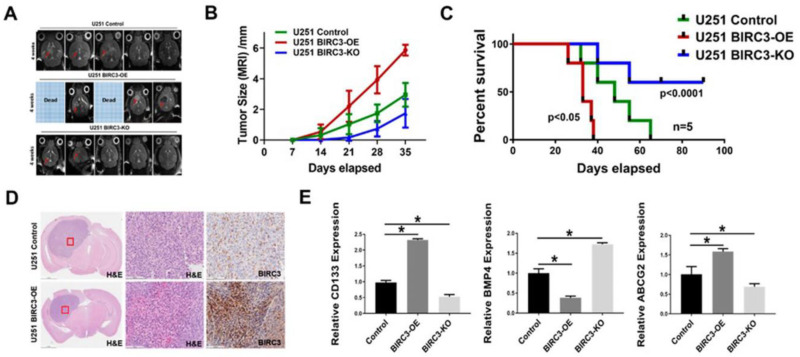
BIRC3 influences tumor initiation and progression in GBM orthotopic xenograft model. From paper by Wu et al., [6]. (**A**). Horizontal axial MRI scan of mouse brain tumors 4 weeks after implantation. Two of BIRC3-OE mice were already dead at 4 weeks. (**B**). Tumor size calculation from MRI scan. *n* = 5. (**C**). Kaplan-Meier survival curve of U251 control BIRC3-OE and BIRC3-KO intracranial injection mice. *n* = 5 mice/group. (**D**). Mice were sacrificed at different timepoints and brain tissues of U251 control and BIRC3-OE groups were fixed in 10% neutral formalin. H&E staining and BIRC3 immunohistochemistry was performed as described in the Material and Methods Section 4. Five mice were included in this histological study and similar results were observed in each animal. (**E**). When mice were sacrificed, part of tumor tissues were isolated. mRNA from tumor tissues were extracted. BMP4, CD133 and ABCG2 mRNA expression analyzed by real-time PCR in extracted tumor tissues. *n* = 3, * *p* < 0.05.
